# Improving the prediction of going concern of Taiwanese listed companies using a hybrid of LASSO with data mining techniques

**DOI:** 10.1186/s40064-016-2186-5

**Published:** 2016-04-27

**Authors:** Yeung-Ja James Goo, Der-Jang Chi, Zong-De Shen

**Affiliations:** Department of Business Administration, National Taipei University, No. 67, Section 3, Ming-Shen East Road, Taipei City, 10478 Taiwan; Department of Accounting, Chinese Culture University, No. 55, Hwa-Kang Road, Yang-Ming-Shan, Taipei City, 11114 Taiwan

**Keywords:** Going concern prediction, Least absolute shrinkage and selection operator (LASSO), Data mining, Neural network (NN), Classification and regression tree (CART), Support vector machine (SVM)

## Abstract

The purpose of this study is to establish rigorous and reliable going concern doubt (GCD) prediction models. This study first uses the least absolute shrinkage and selection operator (LASSO) to select variables and then applies data mining techniques to establish prediction models, such as neural network (NN), classification and regression tree (CART), and support vector machine (SVM). The samples of this study include 48 GCD listed companies and 124 NGCD (non-GCD) listed companies from 2002 to 2013 in the TEJ database. We conduct fivefold cross validation in order to identify the prediction accuracy. According to the empirical results, the prediction accuracy of the LASSO–NN model is 88.96 % (Type I error rate is 12.22 %; Type II error rate is 7.50 %), the prediction accuracy of the LASSO–CART model is 88.75 % (Type I error rate is 13.61 %; Type II error rate is 14.17 %), and the prediction accuracy of the LASSO–SVM model is 89.79 % (Type I error rate is 10.00 %; Type II error rate is 15.83 %).

## Background

Business bankruptcy has caused a huge loss of wealth on the part of investors. Hence, building a valid going concern problem forecast model for an enterprise has become an important goal for both academics and financial practitioners. The high association between going concern doubts (GCD) and business bankruptcy has been verified by past studies (Behn et al. 2001; Geiger and Rama 2003; Koh and Low 2004; Martens et al. 2008; Mokhatab et al. 2011; Yeh et al. 2014). Moreover, the Statement of Auditing Standard (SAS) demands that when an auditor suspects the auditee’s capability of going concern, the auditor should conduct the necessary and reasonable auditing processes required to examine the auditee’s related financial information. If an auditor makes a misjudgment during the auditing process and issues an incorrect audit report, then this has important consequences (e.g. business crisis or investment losses). As a result, the question of how to help auditors notice signs of going concern is an important one.

GCD and bankruptcy forecasts have over the past decade become recognizable with classification problems. Generally, the classification problem carries out a computation in light of the numerical value of some given classification data in order to acquire the relevant classification rule for every classification, bringing unknown classification data into the rule in order to acquire the final classification result. Many going concern prediction (GCP) studies have applied neural network (NN) to build classification models and to acquire results for going concern (GC) issues (Chen and Church 1992; Cornier et al. 1995; Mutchler et al. 1997; Foster et al. 1998; Carcello and Neal 2000; Gaganis et al. 2007; Chen and Lee 2015).

In terms of statistical tools used to handle mega data analysis, machine learning has risen sharply in recent years. It identifies unknown information from complex data and aims to recognize data in order to draw an inference from the structured model, which can act as a reference amount when making decisions for different purposes that are often related to GC issues (Lenard et al. 1995; Anandarajan and Anandarajan 1999; Brabazon and Keenan 2004; Gaganis et al. 2007, Martens et al. 2008; Kirkos et al. 2007a, b; Mokhatab et al. 2011; Salehi and Fard 2013; Yeh et al. 2014; Chen and Lee 2015). The classification method is used most often in these studies, and its results are able to serve as the basis for both decisions and forecasts. However, whether any of the machine learning algorithms in GCP studies is more suitable to this task than another method remains disputed.

Aside from accuracy of the prediction models, the occurrence of Type I error and Type II error cannot be ignored (O’Leary 1998; Kirkos et al. 2007a, b; Tasi and Huang 2010; Chen et al. 2015). A Type II error may especially cause damages and high costs. If an auditor issues a wrong audit report due to his/her misjudgment, then it affects not only the enterprise and stakeholders, but also many investors. Moreover, the CPA may be sued. The costs for Type II errors are rather severe in the U.S. Examples include the Enron scandal in 2001 (Benston and Hartgraves 2002) and WorldCom fraud in 2003. Taiwan has had its own financial fraud cases for Procomp Informatics and Infodisc in 2004 and Summit Computer in 2006.

The purpose of this study is to develop a satisfactory model for forecasting the GCD of firms and to forecast an omen for such GCD and to reduce damage to both investors and auditors. This study applies support vector machine (SVM), as well as the classification and regression trees (CARTs) in the machine learning method, as its basis and matches LASSO in order to separately establish a classification model and draw up a comparison.

## Literature review

### Going concern concept and reports

Before investors invest in a company, they should understand the viability of the company. This kind of viability relates to the ability of management to properly manage the company’s overall resources in order to survive. In uncertain situations, investors expect auditors to provide early warnings of business failure and risks of bankruptcy (Chen and Church 1996).

Pursuant to the provision of SAS No. 59, an auditor’s consideration of an entity’s ability to continue as GC requires an explicit evaluation of the auditee’s continued viability during the audit process. As a result, the GCD report is used as a warning sign when an auditor suspects an auditee’s weakness in terms of GCD (Lenard et al. 1995).

### Criteria for issuing an audit report by CPA for going concern

Taiwan’s auditing standards bulletin No. 16 stipulates that the compilation of financial statements is often based on an assumption of going concern. It further requires that auditors shall comply with the stipulations as specified in the bulletin when they evaluate reasonable assumptions of going concern. CPAs are able to issue unqualified opinion audit reports if they eliminate their doubt about the ability of going concern after evaluating the rationality of the assumption of going concern. If CPAs consider the auditee’s future measures are reasonable and necessary to be disclosed in the financial report, then a qualified opinion audit report or an adverse opinion audit report is needed. If the CPA cannot eliminate doubts about the auditee’s ability of going concern, but the auditee’s financial statements have been disclosed, then the CPA shall issue an unqualified-modified opinion audit report. If the auditee’s financial statements have not been properly disclosed, then the CPA shall issue a qualified opinion audit report or an adverse opinion audit report depending on the significance. If a CPA has confirmed that the assumption of going concern for the compilation of financial statements is not consistent with the actual situation and would have serious consequences, then the CPA shall issue an adverse audit opinion report. If the CPA cannot eliminate doubt, or the assumption is not consistent with the actual situation, then explanatory notes should be included in the audit report, and these notes should form the audit report (Auditing Standards Board of the Republic of China Accounting Research Development Foundation, Auditing standard bulletin and auditing practice, 2013).

### Traditional classification studies

The GCP model carries out a computation that mainly depends on the numerical values of train subset data of financial and non-financial indicators in order to acquire the relevant classification rule for every classification and brings data subsets into the rule in order to acquire the final classification result.

Based on the difficulty of the GCD assessment, many authors apply LR in order to make a GCP classification in relation to the GC issue (Chen and Church 1992; Cornier et al. 1995; Mutchler et al. 1997; Foster et al. 1998; Carcello and Neal 2000; Gaganis et al. 2007). However, the traditional classification method suffers from the limitation of having to be in accordance with specific assumptions in the data.

### Machine learning classification methods

The machine learning approach has often been adopted in the literature. Many studies have attempted to apply the machine learning approach as a base to build a classification model. These studies point out that adopting this method leads to outstanding prediction accuracy. Several studies applying a machine learning approach (e.g. SVM, DT, NN, etc.) to GCD, indicating that these approaches are able to forecast the GC status of businesses and provide useful financial data for the GC issue (Brabazon and Keenan 2004; Koh and Low 2004; Martens et al. 2008; Mokhatab et al. 2011; Salehi and Fard 2013; Yeh et al. 2014).

On a similar classification issue, Tasi and Wu (2008) apply NN in relation to bankruptcy predictions and credit scores. Chen et al. (2014) employ DT, SVM, and LR in the Fraudulent Financial Statements forecast in order to acquire excellent classification results. Based on these studies, this study utilizes the aforementioned LR, SVM, NN, and DT approaches as the basis upon which to build a classification model.

## Methods

The purpose of this study is to establish a two-stage going concern doubt prediction model that integrates financial and non-financial indicators. The process of this study creates a least absolute shrinkage and selection operator (LASSO) to obtain the results for important indicators of GCD after screening. For forecast modeling, the classification approach includes the following machine learning techniques: NN, DT, and SVM. Finally, this study draws a comparison and conducts an analysis in order to obtain better GC prediction results.

### Least absolute shrinkage and selection operator (LASSO)

Stepwise regression has been applied in related work in the past, but there are significant problems with stepwise methods, which have been admirably summarized by Harrell (2001). These problems are as follows: (1) R^2^ values are biased. (2) The F test statistics do not have the claimed distribution. (3) The standard errors of the parameter estimates are too small. (4) Consequently, the confidence intervals around the parameter estimates are too narrow. (5) The parameter estimates are highly biased in absolute value. (6) Collinearity problems are exacerbated.

This study applies LASSO as a feature selection method, which was first proposed by Tibshirani (1996). This algorithm minimizes the residual sum of squares subject to the sum of the absolute values of the coefficient being less than a constant.

1 $$ \mathop {\hat{\beta }^{L} }\limits_{\sim} = \arg \hbox{min} \left\{ {\sum\limits_{i = 1}^{N} {\left( {y_{1} - \alpha - \sum\limits_{j} {\beta_{j} \chi_{ij} } } \right)^{2} } } \right\} $$

2 $$ \begin{aligned} & {\text{subject to}} \\ & \sum\limits_{j = 1}^{p} {\left| {\hat{\beta }_{j}^{L} } \right|} \le (Constant) \\ \end{aligned} $$

If $t > \sum\nolimits_{j = 1}^{p} {\left| {\hat{\beta }_{j}^{0} } \right|} ,$ then the LASSO algorithm yields the same estimate as the OLS estimate.

However, if $0 < t < \sum\nolimits_{j = 1}^{p} {\left| {\hat{\beta }_{j}^{0} } \right|} ,$ then the problem is equivalent to:

3 $$ \mathop {\hat{\beta }^{L} }\limits_{\sim} = \arg \hbox{min} \left[ {\sum\limits_{i = 1}^{N} {\left( {y_{1} - \alpha - \sum\limits_{j} {\beta_{j} \chi_{ij} } } \right)^{2} + \lambda \sum\limits_{j} {\left| {\beta_{j} } \right|} } } \right] $$

where, λ > 0. We shall show later that the relation between λ and the LASSO parameter t is one-to-one.

Due to the nature of the constraint, LASSO tends to produce some coefficients that are exactly zero. Compared to OLS, whose predicted coefficient $\mathop {\beta^{0} }\limits_{\sim}$ is an unbiased estimator of $\mathop \beta \limits_{\sim} ,$ both ridge regression and LASSO sacrifice a little bias in order to reduce the variance of the predicted values and improve the overall prediction accuracy. In this past decade, LASSO has been widely applied in many different ways and variants (Tibshirani et al. 2005; Colombani et al. 2013; Yamada et al. 2014; Toiviainen et al. 2014; Connor et al. 2015).

### Neural networks (NN)

**Fig. 1:**
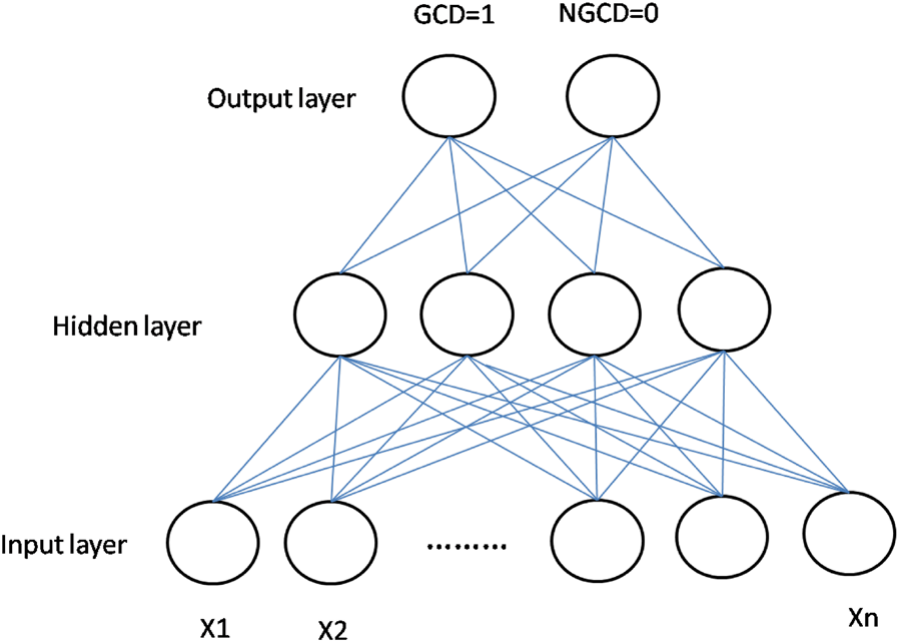
Neural network model

Neural networks refer to information processing systems that simulate bio-neural networks. They use a large number of connected artificial neurons in order to simulate the capacity of neural networks (Anandarajan and Anandarajan 1999; Tasi and Wu 2008; Korol 2013; Chen et al. 2015). Since NN is equipped with the functions of high-speed calculation and information de-noises, it is capable of solving many sophisticated classification and forecasting issues. The most common NN model has three layers: input layer, hidden layer, and output layer. The input layer is used to receive variables. The hidden layer is constituted by neutrons, and its major purpose is to increase the complexity of neural networks, so that they can simulate complicated linear relations. The output layer generates post-processing prediction results. The three layers of the NN model are illustrated in Fig. 1.

The MLP network is a function of one or more predictors that minimizes the prediction error of one or more targets. Predictors and targets can be a mix of categorical and continuous fields. The general architecture for MLP networks can be described as:

4 $$ {\text{Input layer:}}\;J_{0} = P\;{\text{units,}}\;a_{0:1, \ldots ,} a_{0:j0} ;{\text{ with}}\;a_{0:j0} = x_{j} $$

5 $$ {\text{ith hidden layer:}}\;J_{i} \;{\text{units,}}\;a_{i:1, \ldots ,} a_{{i:J_{i} }} ;\;{\text{with}}\;a_{i:k} = \gamma_{i} (c_{i:k} )\;{\text{and}}\;c_{i:k} = \sum\limits_{j = 0}^{{J_{1} }} {_{{w_{i:j,k} a_{i - 1:j} }} } ,\;{\text{and}}\;a_{i - 1:0} = 1 $$

6 $$ {\text{Output layer:}}\;J_{I} = R\;{\text{units,}}\;a_{I:1, \ldots ,} a_{{I:J_{i} }} ;{\text{ with}}\;a_{I:k} = \gamma_{I} (c_{I:k} )\;{\text{and}}\;c_{I:k}^{m} = \sum\limits_{j = 0}^{{J_{1} }} {_{{w_{I:j,k} a_{i - 1:j} }} } ,\;{\text{and}}\;a_{i - 1:0} = 1 $$

The training finally proceeds through at least one complete pass of the data. The search should then be stopped according to the stopping criteria.

Where, $$ X(m) = x_{1, \ldots ,}^{(m)} x_{p}^{(m)} $$ is the input vector; pattern *m*, *m* = 1, … M; $$ Y(m) = y_{1, \ldots ,}^{(m)} y_{R}^{(m)} $$ is the target vector; pattern *m*; *I* is the number of layers, discounting the input layer; *J*_*i*_ is the number of units in layer *i*; $$ J_{0} = P,J_{i} = R, $$ discounting the bias unit; $$ \Gamma ^{c} $$ and $$ \Gamma $$ are a set of categorical outputs and continuous outputs; $$ \Gamma _{h} $$ is a set of sub-vectors of $$ Y^{(m)} $$ containing 1-of c coded hth categorical field; and $$ w_{i:j,k} $$ is a weight leading from layer *i* − 1, unit *j* to layer *i*, unit *k*. No weights connect $$ a_{i - 1:j}^{m} $$ and the bias $$ a_{i:0}^{m} $$—that is, there is no $$ w_{i:j,0} $$ for any *j*. Finally, $$ c_{i:k}^{m} $$ is $$ \sum\nolimits_{j = 0}^{{J_{i} - 1}} {w_{i:j,k} a_{i - 1:j}^{m} ,i = 1, \ldots ,I} $$ and $$ \gamma_{i} (c) $$ is an activation function for layer *i*.

### Support vector machine (SVM)

Support vector machine (SVM) was developed by Boser et al. (1992) to provide better solutions than other traditional classifiers, such as neural networks. SVM is a type of maximal margin classifier, in which the classification problem can be represented as an optimization process, which finds the maximum-margin hyper-plane from a given training dataset D as described by:

7 $$ D = \left\{ {(x_{i} ,y_{i} )\left| {x_{i} \in {\mathbb{R}}^{p} ,y_{i} \in \{ - 1,1\} } \right.} \right\}_{i = 1}^{n} $$

where $y_{i}$ is either 0 or 1, and n is the number of training data. Each $x_{i}$ is a p-dimensional vector having the feature quantity ${\mathbb{R}}.$ Any hyper-plane can be written as:

8 $$ w \cdot x - b = 0 $$

where, w is the vector to the hyper-plane. If the training data are linearly separable, then the hyper-plane can be described as:

9 $$ w \cdot x - b = 1\;{\text{and}}\;w \cdot x - b = - 1 $$

The distance between these two hyper-planes is $2 /\left\| w \right\|,$ and so the purpose is to minimize w. Therefore, the algorithm can be rewritten as:

10 $$ {\text{Minimize:}}\;\left\| w \right\|,{\text{ under the condition of}}\;y_{i} (w \cdot x_{i} - b) \ge 1,{\text{ for any}}\; 1\le {\text{i}} \le {\text{n}} $$

We can also reformulate the equation without changing the solution as:

11 $$ {\mathop{\arg \min}\limits_{(w,b)}} \frac{1}{2}\left\| w \right\|^{2} ,\,{\text{under the condition of}}\;y_{i} (w \cdot x_{i} - b) \ge 1,{\text{ for any}}\;1 \le {\text{i}} \le {\text{n}} $$

The hyper-plane, or a set of hyper-planes, can be used as the separate lines in a classification. The SVM approach has recently been used in several financial applications (Martens et al. 2008; Tasi 2008; Li and Sun 2009; Chen et al. 2014; Yeh et al. 2010, 2014).

### Class and regression tree (CART)

Classification and regression tree (CART) is a flexible method to describe how the variable Y is distributed after assigning the forecast vector X (Patil et al. 2012). It is able to classify huge amounts of data according to the division rule so as to identify valid data and thereby achieve ideal results (Kirkos et al. 2007a, b; Salehi and Fard 2013; Kim and Upneja 2014; Marsala and Petturiti 2015). CART uses the binary tree to divide the forecast space into certain subsets on which the target variable distribution is continuously even. The “leaf” nodes correspond to different division areas that are determined by Splitting Rules relating to each internal node. By moving from the tree root to the leaf node, any forecast sample will be given only a leaf node.

This algorithm uses the GINI Index to determine in which attribute the branch should be generated. The building process of the model is to choose the attribute whose GINI index is a minimum after splitting. It can be described as:

12 $$ GINI(T) = 1 - \sum\limits_{i = 1}^{m} {P_{i}^{2} } $$

Let X be divided into *n* subsets, $\{ T1,T2, \ldots Tn\} .$ Among them, T_i_’s sample number is n_i_. Thus, the Gini index divided according to property X is described as:

13 $$ GINI(T) = 1 - \sum\limits_{i = 1}^{n} {\frac{{n_{i} }}{n}GINI(T_{i} )} $$

CART divides the property that leads a minimum value after the division.

## Empirical analysis

### Data collection and sampling

Research samples are drawn from GCD and NGCD firms in Taiwan from 2002 to 2013. 48 GCD firms are selected from all the listed companies of the Taiwan Economic Journal (TEJ) Data Bank. We adopt the 1-by-3 pair technique in order to match 144 NGCD firms. Thus, there are 192 firms in total that serve as our research sample of GCD and NGCD firms as shown in Table 1. Based on the indicators’ selection in prior studies on GCD (Anandarajan and Anandarajan 1999; Behn et al. 2001; Kirkos et al. 2007a, b; Martens et al. 2008; Yeh et al. 2014), we prepare a set of 22 variables, as displayed in Table 2. These indicators are available in the TEJ database.

**Table 1:** Samples

Year	2002	2003	2004	2005	2006	2007	2008	2009	2010	2011	2012	2013	Total
GCD samples	20	2	4	4	4	1	4	2	2	1	2	2	48
NGCD samples	60	6	12	12	12	3	12	6	6	3	6	6	144

**Table 2:** Research variables

No.	Variable description/Definition or formula	Sources
X1	Total assets: Natural logarithm of total assets	Zhou et al. (2012), Chen et al. (2014), Yeh et al. (2014) and Chen and Lee (2015)
X2	Net sales: Natural logarithm of net sales	Tang and Firth (2011) and Chen et al. (2014)
X3	Current ratio: Current assets/Current liabilities	Lin (2009), Huang and Lu (2000), Sun et al. (2011), Zhou et al. (2012), Yeh et al. (2014), Chen and Lee (2015) and Chen et al. (2015)
X4	Debt ratio: Total liabilities/Total assets	Lin (2009), Huang and Lu (2000), Yeh et al. (2010), Jiang and Habib (2012), Chen et al. (2014, 2015), Yeh et al. (2014) and Chen and Lee (2015)
X5	Current assets: Natural logarithm of current assets	Korol (2013)
X6	Undistributed surplus: Natural logarithm of undistributed surplus	Chen and Lee (2015)
X7	Long term liabilities: Natural logarithm of long term liabilities	Korol (2013)
X8	Inventory: Natural logarithm of inventory	Salehi and Fard (2013)
X9	Total equity: Natural logarithm of total equity	Korol (2013)
X10	Total liabilities: Natural logarithm of total liabilities	Chen and Lee (2015)
X11	Net profit before tax: Income before tax	Chen et al. (2015)
X12	Operating cash flow: Cash flow from operating activities	Jiang and Habib (2012) and Chen et al. (2015)
X13	Accounts receivable turnover: Net sales/Average accounts receivable	Sun and Li (2008), Huang and Lu (2000), Yeh et al. (2010), Chen and Lee (2015) and Chen et al. (2015)
X14	Inventory turnover: Cost of goods sold/Average inventory	Zhou et al. (2012), Chen and Lee (2015) and Chen et al. (2015)
X15	Stockholding ratio of directors and supervisors: Number of stocks held by directors and supervisors/Total number of common stock outstanding	Chen and Lee (2015) and Chen et al. (2015)
X16	Big CPA firm or not (Big 4 in Taiwan): 1 for companies audited by BIG4, otherwise is 0	Jiang and Habib (2012), Yeh et al. (2014), Chen and Lee (2015) and Chen et al. (2015)
X17	Change CPA firm (CPA) or not: 1 is for change; 0 is for non-change	Anandarajan and Anandarajan (1999), Yeh et al. (2014) and Chen and Lee (2015)
X18	Current liabilities: Natural logarithm of current liabilities	Salehi and Fard (2013)
X19	Operating income: Natural logarithm of operating income	Salehi and Fard (2013) and Chen et al. (2015)
X20	Total assets turnover: Net Sales/Average total assets	Sun and Li (2008) and Sun et al. (2011)
X21	Earnings before interest and tax (EBIT)	Salehi and Fard (2013) and Chen et al. (2015)
X22	Return on assets (ROA): [Net income + interest expense × (1–tax rate)]/Average total assets	Martens et al. (2008), Lin (2009), Sun et al. (2011), Zhou et al. (2012), Jiang and Habib (2012) and Chen et al. (2015)

For the consideration of the number of samples, in order to avoid having too few samples in the test group and in order to improve test accuracy, we randomly gather 5 subsets from our original sample set and conduct fivefold cross validation.

### Model development

This study begins by reducing the indicators using the LASSO screening method. The variables screened serve as the input variables for NN, CART and SVM. Next, the study carries out the model training and testing with every method. Finally, the study compares the merits and demerits of the classification ratio and provides relevant suggestions based on the analytic results.

**Fig. 2:**
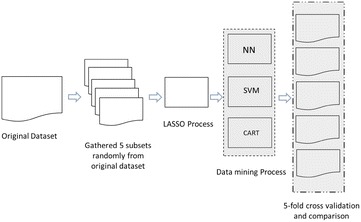
Research process

Model construction is divided into three parts. The first part is replacement sampling; the second part is the LASSO feature selection; and the third part compares the test results of four kinds of classification models. The research process of this study is shown in Fig. 2.

### Important variable screening

While constructing the classification model, many variables may be included, but not all of these variables are actually important. Therefore, unimportant variables need to be eliminated in order to construct a simpler classification model. There is quite a number of ways to screen variables, of which the LASSO algorithm has shown excellent performance in reducing variables (Connor et al. 2015).

**Fig. 3:**
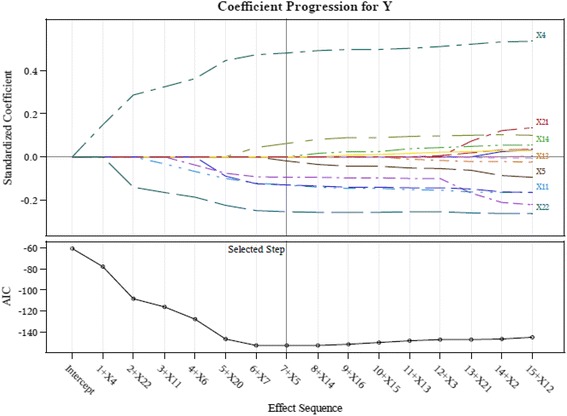
LASSO variables screening process Work-Group 1

**Fig. 4:**
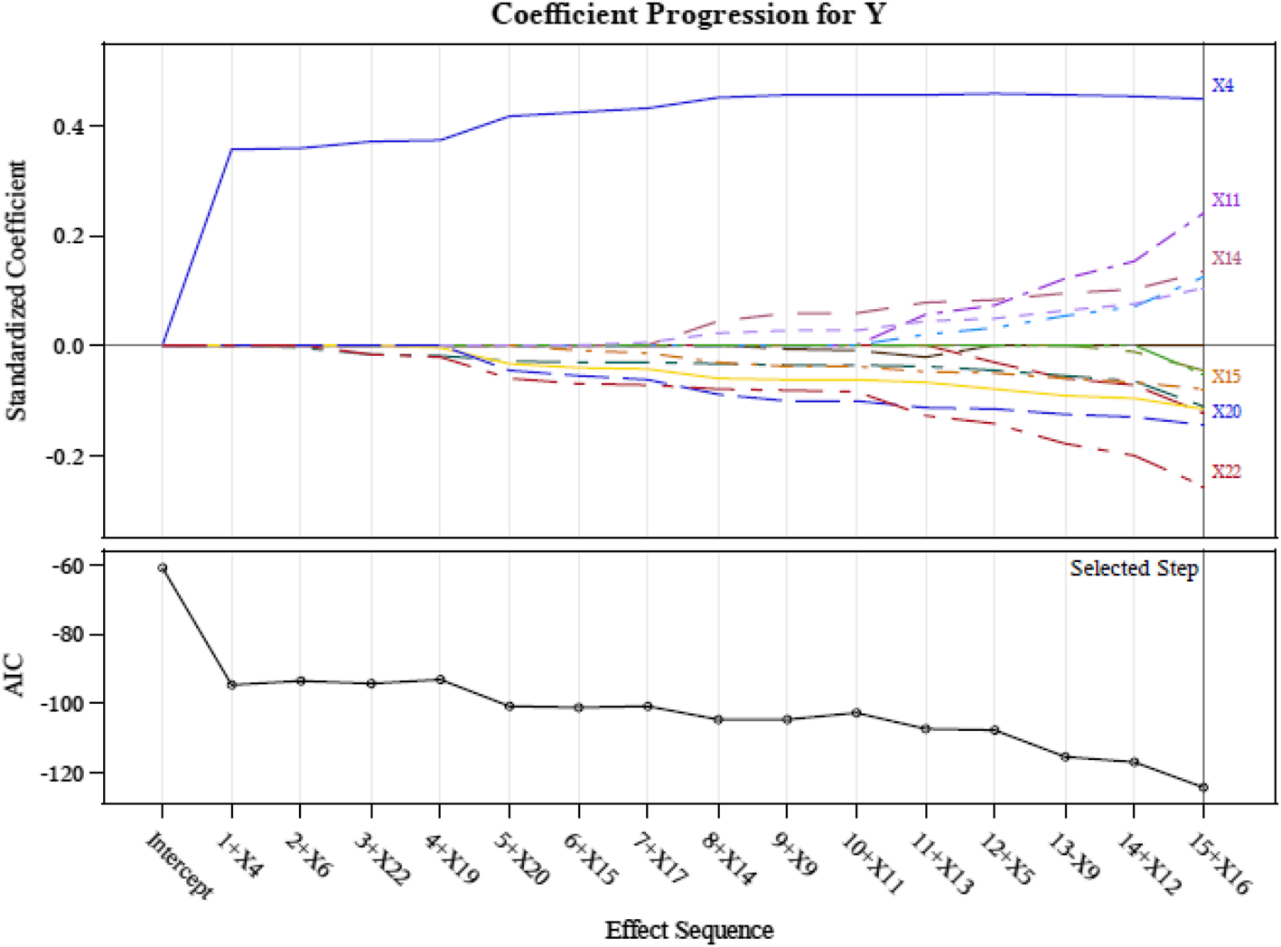
LASSO variables screening process Work-Group 2

**Fig. 5:**
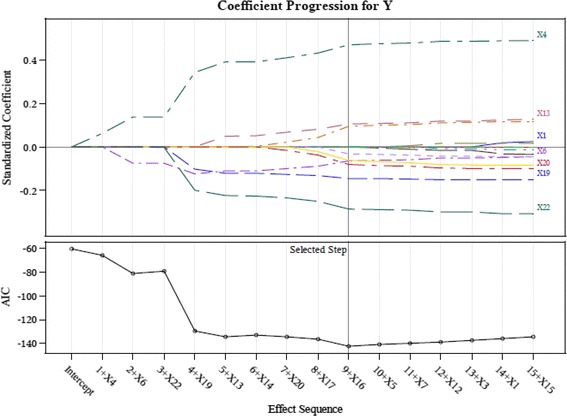
LASSO variables screening process Work-Group 3

**Fig. 6:**
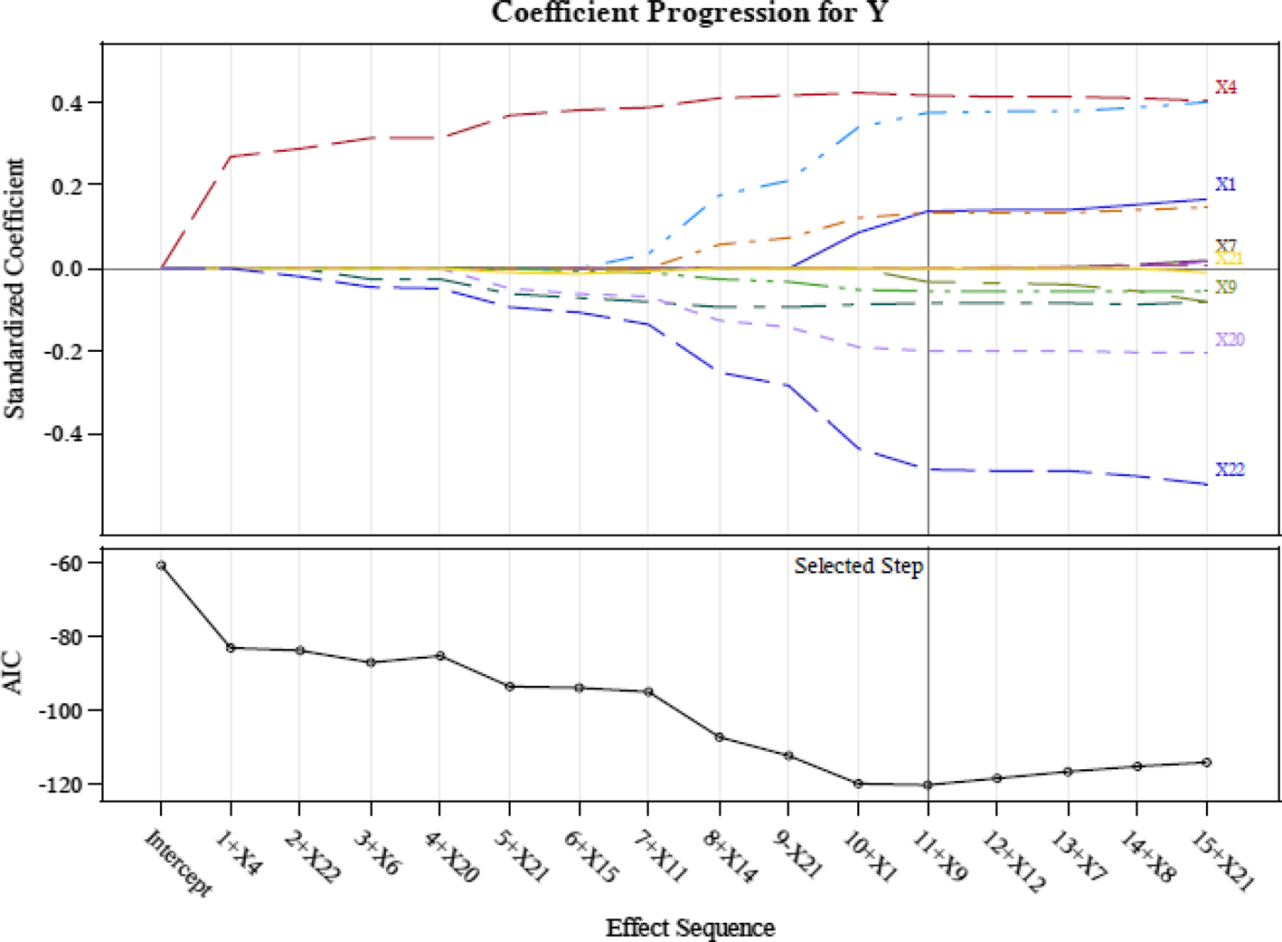
LASSO variables screening process Work-Group 4

**Fig. 7:**
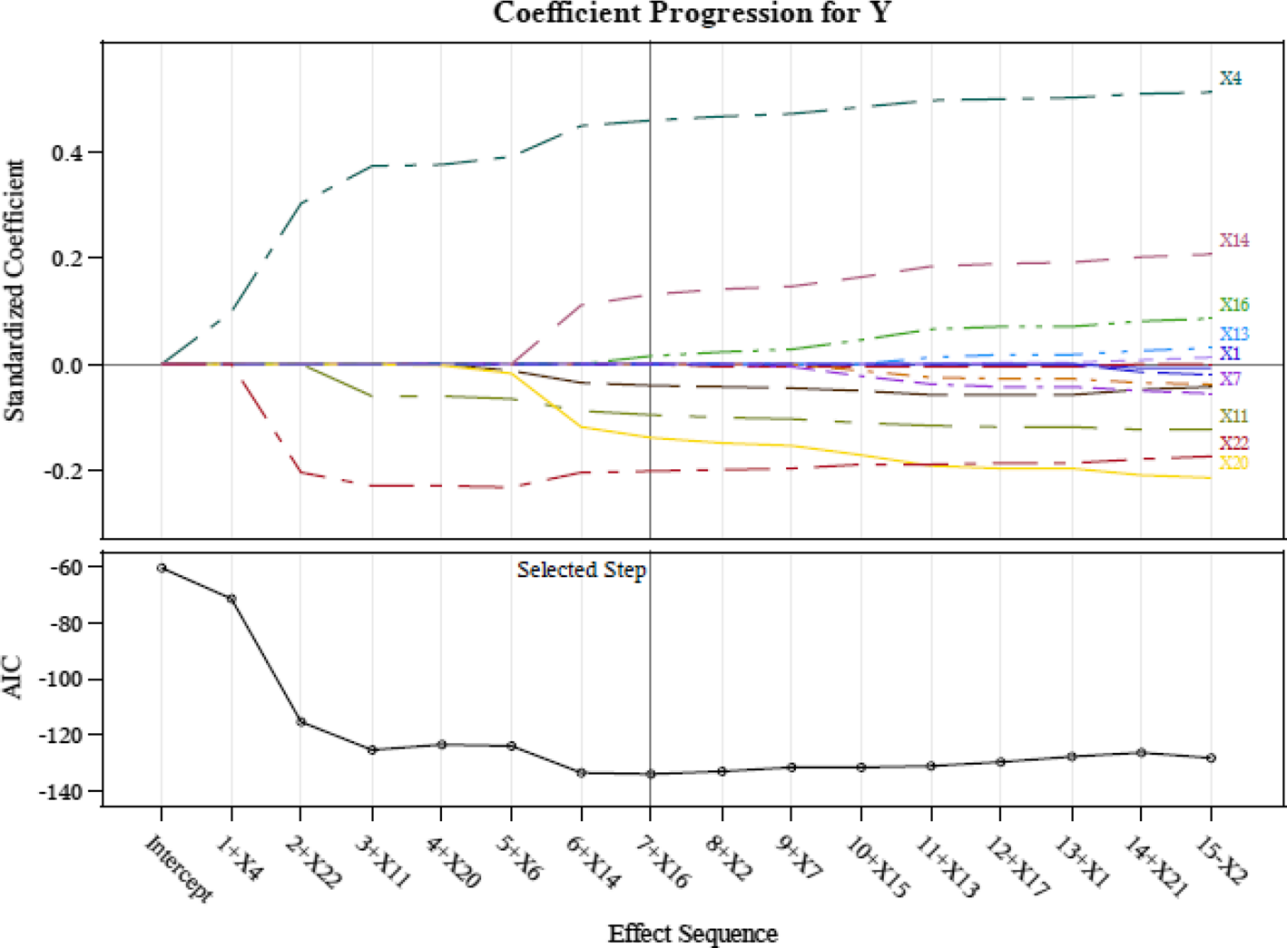
LASSO variables screening process Work-Group 5

This study therefore adopts the suggestions of Connor et al. (2015) and screens the important indicators using the LASSO technique in order to retain only input variables with a significant influence. We employ the LASSO available in the SAS software to calculate the AIC values and coefficients of variable importance. The input variables of the study are screened using LASSO to acquire the results shown in Table 3 and Figs. 3, 4, 5, 6 and 7.

**Table 3:** LASSO variables’ screening process

Steps	Work-G1 (AIC)	Work-G2^a^ (AIC)	Work-G3 (AIC)	Work-G4^b^ (AIC)	Work-G5 (AIC)
1	X4 (−77.5676)	X4 (−94.7118)	X4 (−66.0500)	X4 (−83.1760)	X4 (−71.2937)
2	X22 (−108.2326)	X6 (−93.3790)	X6 (−80.9976)	X22 (−83.9267)	X22 (−115.3547)
3	X11 (−116.1226)	X22 (−94.0645)	X22 (−79.4015)	X6 (−87.0297)	X11 (−125.4222)
4	X6 (−127.3604)	X19 (−93.0137)	X19 (−129.3612)	X20 (−85.2646)	X20 (−123.5628)
5	X20 (−146.4499)	X20 (−100.9320)	X13 (−134.4688)	X15 (−94.1284)	X6 (−124.3376)
6	X7 (−152.5126)	X15 (−101.0658)	X14 (−132.8479)	X11 (−95.2185)	X14 (−133.9785)
7	X5 (−152.5561)	X17 (−100.642)	X20 (−134.1510)	X14 (−107.4634)	X16 (−134.0137)
8		X14 (−104.7244)	X17 (−136.4395)	X1 (−120.0362)	
9		X11 (−102.8433)	X16 (−142.2861)	X9 (−120.4143)	
10		X13 (−107.1809)			
11		X5 (−107.8717)			
12		X12 (−116.8996)			
13		X16 (−124.2823)			

^a^X9 effect entered at step, AIC value is −104.7244, removed at step 13, AIC value form −107.8717 decease to −115.5186

^b^X21 effect entered at step 5, AIC value is −93.7699, removed at step 9, AIC value form −107.4634 decease to −112.5140

This study proposes a GCD prediction model for CPAs. Thus, the study adopts the indicators as input variables, which were selected in each screening process (Work-Groups 1–5). The important variables selected by using LASSO include: X4 (Debt ratio), X6 (Undistributed surplus), X20 (Total assets turnover), and X22 (Return on assets; ROA).

X4 (Debt ratio: Total liabilities/Total assets) is an important measure of the debt ratio and capital structure of a company. Generally, capital is sourced from stockholders or external financing. Financing has a leverage that can increase the return on investment. Moreover, interest costs are not taxed, and thus financing has numerous advantages, but if debt is high, then financial leverage may increase risk. If a firm’s operations are not as good as expected, then bankruptcy may occur. X6 (Undistributed surplus) is net income after withdrawal of legal and special surplus and can be used to pay cash dividends, expansion, or R&D. X20 (Total assets turnover: Net Sales/Average total assets) is an important measure to evaluate the operation quality of corporate assets and utilization efficiency. The greater the turnover rate is, the faster the turnover of total assets, and the stronger the sales ability. X22 (Return on assets (ROA): [Net income + interest expense × (1 − tax rate)]/Average total assets) shows the percentage of how profitable a company’s assets are in generating revenue.

This study subsequently takes the 4 variables above as new input predictors in order to construct a prediction/classification model. The descriptive statistics and correlation of input variables are shown as Tables 4 and 5.

**Table 4:** Descriptive statistics of input variables

Variable		N	Mean	SD	Min	Max
X4	Debt ratio	192	51.0965	21.6263	4.8700	101.9700
X6	Undistributed surplus	192	−346,749.52	2,210,187.98	−22,801,544.00	5,561,297.0000
X20	Total assets turnover	192	0.8593	0.6895	0.0300	4.8400
X22	Return on assets (ROA)	192	−0.0756	0.2762	−2.0997	0.3695

**Table 5:** Correlation of input variables

Input variable		X4	X6	X20	X22
X4	Debt ratio	1	–	–	–
X6	Undistributed surplus	−0.3137	1	–	–
		<0.0001			
X20	Total assets turnover	0.0048	0.2430	1	–
		0.9478	0.0007		
X22	Return on assets (ROA)	−0.2752	0.2146	0.1941	1
		0.0001	0.0028	0.0070	

### Classification model

This study employs IBM SPSS modeler 14.0 to build classification models NN, CART, and SVM. The cross-validation results of the training and testing subsets are shown as Tables 6, 7 and 8.

**Table 6:** LASSO–NN model—the fivefold cross validation results

Subset	Training set					Testing set				
	Predicted group		Hit ratio (%)	Type I error (%)	Type II error (%)	Predicted group		Hit ratio (%)	Type I error (%)	Type II error (%)
1	71	1	98.96	1.39	0.00	70	2	94.79	2.78	12.50
	0	24				3	21			
2	70	2	90.62	2.78	29.17	60	12	85.42	16.67	8.33
	7	17				2	22			
3	69	3	92.71	4.17	4.17	64	8	90.62	11.11	4.17
	1	23				1	23			
4	60	12	87.50	16.67	12.50	59	13	85.42	18.06	4.17
	3	21				1	23			
5	70	2	96.88	2.78	4.17	63	9	88.54	12.50	8.33
	1	23				2	22			
Avg.			93.33	5.56	10.00			88.96	12.22	7.50

**Table 7:** LASSO–CART model—the fivefold cross validation results

Subset	Training set					Testing set				
	Predicted group		Hit ratio (%)	Type I error (%)	Type II error (%)	Predicted group		Hit ratio (%)	Type I error (%)	Type II error (%)
1	66	6	93.75	8.33	0.00	68	4	93.75	5.56	8.33
	0	24				2	22			
2	70	2	93.75	2.78	16.67	57	15	86.46	20.83	16.67
	4	20				4	20			
3	67	5	92.71	6.94	8.33	65	7	90.62	9.72	20.83
	2	22				5	19			
4	69	3	92.71	4.17	16.67	60	12	83.33	16.67	12.50
	4	20				3	21			
5	72	0	94.79	0.00	20.83	61	11	89.58	15.28	12.50
	5	19				3	21			
Avg.			93.54	4.44	12.50			88.75	13.61	14.17

**Table 8:** LASSO–SVM model—the fivefold cross validation results

Subset	Training set					Testing set				
	Predicted group		Hit ratio (%)	Type I error (%)	Type II error (%)	Predicted group		Hit ratio (%)	Type I error (%)	Type II error (%)
1	71	1	96.88	1.39	8.33	66	6	91.67	8.33	8.33
	2	22				2	22			
2	70	2	90.62	2.78	29.17	66	6	89.58	8.33	16.67
	7	17				4	20			
3	71	1	92.71	1.39	25.00	66	6	88.54	8.33	20.83
	6	18				5	19			
4	68	4	87.50	5.56	33.33	62	10	86.46	13.89	12.50
	8	16				3	21			
5	72	0	96.88	0.00	12.50	70	2	92.71	2.78	20.83
	3	21				5	19			
Avg.			92.92	2.22	21.67			89.79	10.00	15.83

### LASSO–NN model

The NN model is set as follow: (1) model type is set at Multilayer Perceptron (MLP), one hidden layer, and maximum training cycles stop at 250 times. The LASSO–NN model classification results are shown as Table 6.

**Fig. 8:**
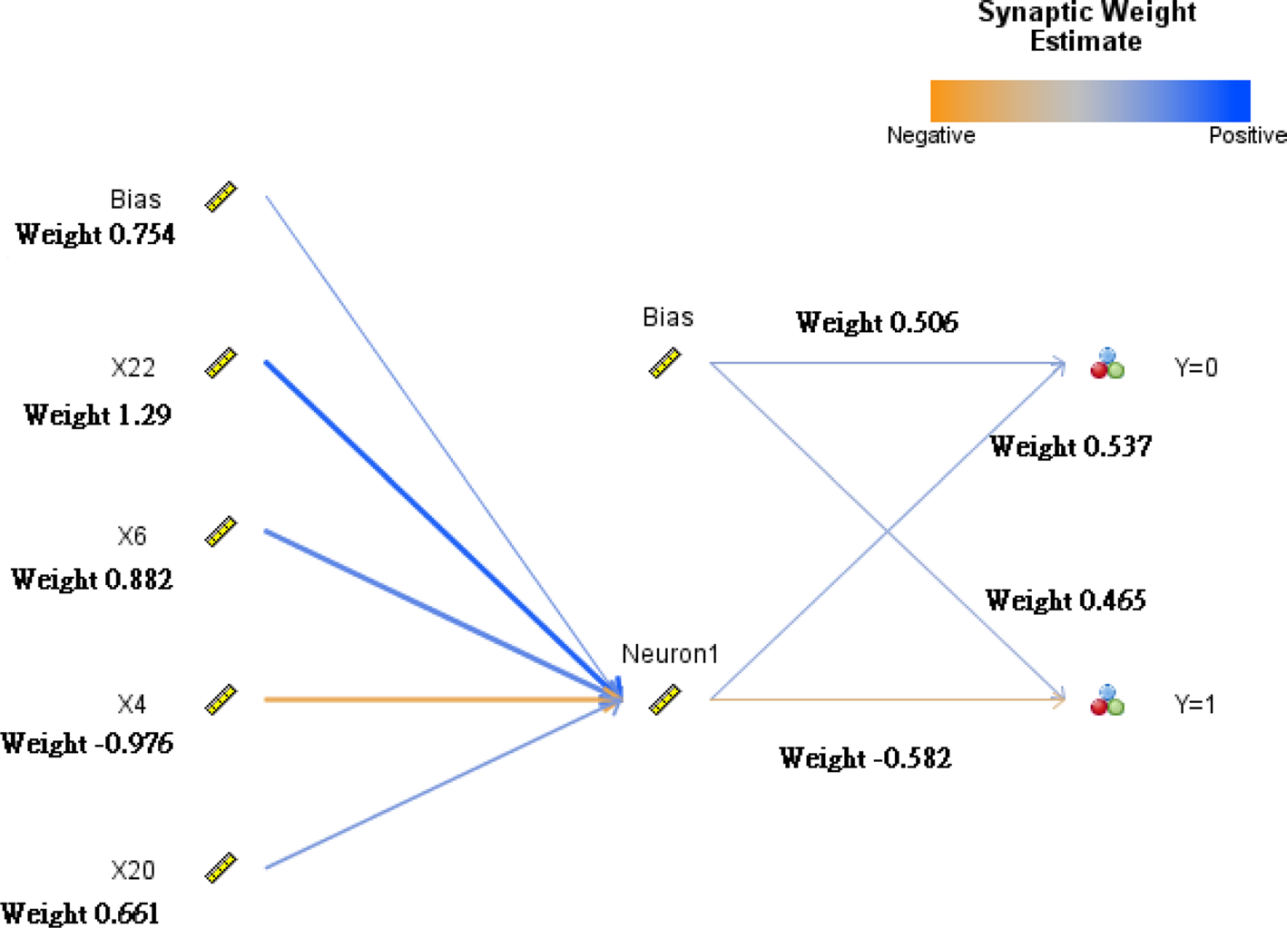
Weight of each node of the NN model

**Fig. 9:**
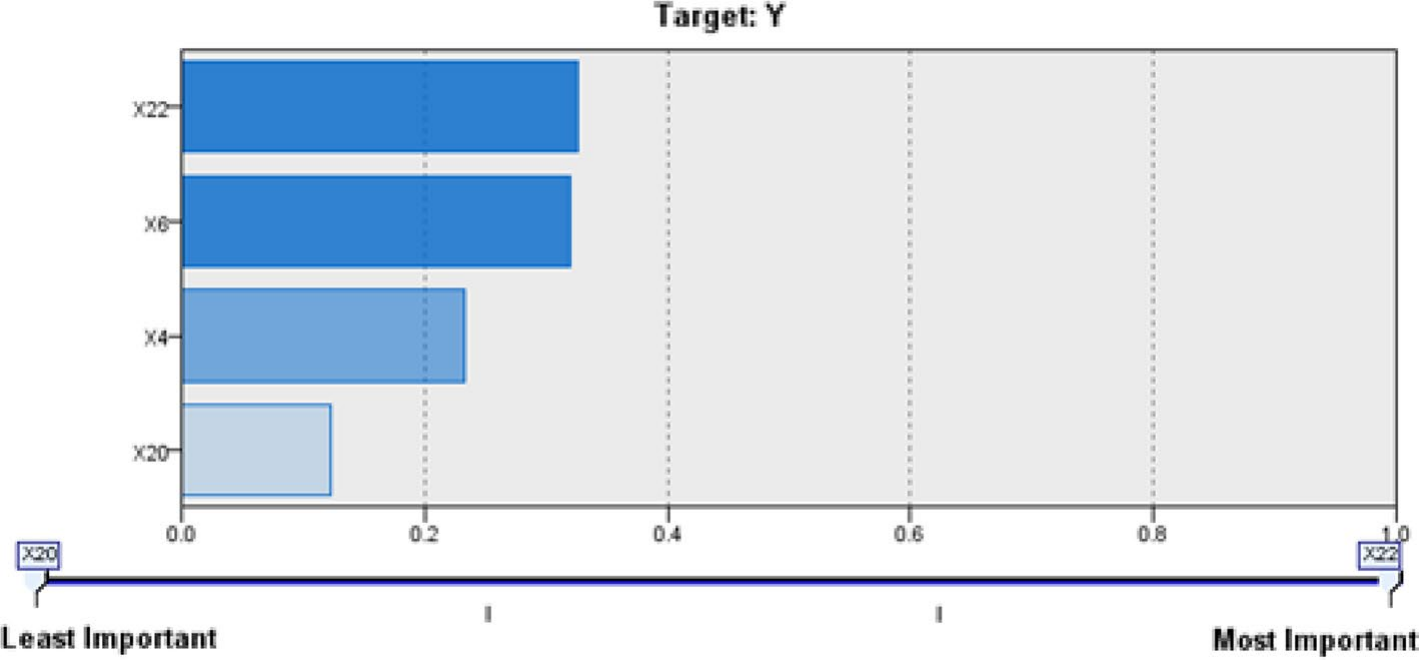
Importance of variables

On average, 9 of the 72 NGCD materials are incorrectly classified, and the Type I error rate is 12.22 %. In addition, 22 of the 24 GCD materials are correctly classified, while the remaining 2 GCD materials are incorrectly classified in NGCD. The Type II error is 7.50 %. The weight of each node and importance of variables are shown as Figs. 8 and 9.

### LASSO–CART model

This study constructs the LASSO–CART model, sets maximum depth at 5, and adopts the Gini index as an impurity measure for categorical targets. The forecast results of the LASSO–CART prediction model are shown in Table 7. On average, 62 of the 72 NGCD materials are correctly classified, while 10 of them are incorrectly classified in GCD, for a Type I error of 13.61 %. On the other hand, 20 of the 24 GCD materials are correctly classified, with the remaining 2 GCD materials incorrectly classified in NGCD. The Type II error is 14.17 %.

### LASSO–SVM model

In terms of the LASSO–SVM model, the kernel type is set at “Linear”, the stopping criteria is set at 1.0E−3, and the regularization parameter is set at 10 and 0.1 of the regression precision.

The LASSO–SVM classification results are shown in Table 8. On average, 66 of the 72 NGCD materials are correctly classified, while 6 of them are incorrectly classified in GCD. The Type I error is 10.00 %. In addition, 20 of the 24 GCD materials are correctly classified, with the remaining 4 GCD materials incorrectly classified in NGCD. The Type II error is 15.83 %.

### Model comparison and statistical test

According to the empirical results (Tables 6, 7, 8), the prediction accuracy of the LASSO–NN model is 88.96 % (Type I error rate is 12.22 %; Type II error rate is 7.50 %), the prediction accuracy of the LASSO–CART model is 88.75 % (Type I error rate is 13.61 %; Type II error rate is 14.17 %), and the prediction accuracy of the LASSO–SVM model is 89.79 % (Type I error rate is 10.00 %; Type II error rate is 15.83 %). Our comparison follows that of Kirkos et al. (2007a, b), Tasi and Huang (2010) and Chen et al. (2014). We not only focus on the hit ratio of the models, but also consider the Type I error and Type II error rates.

Unlike past works, which typically use Type I errors to judge the performance of a forecasting model, GCP studies prefer to use Type II errors to determine the performance of forecasting models. In order to confirm the significant difference between prediction models, this study uses the Wilcoxon two-sample test and the Kruskal–Wallis test, with the results shown in Table 9. The test results reveal a significant difference among the LASSO–NN, LASSO–CART, LASSO–NN, and LASSO–SVM prediction models.

**Table 9:** Statistical tests

Statistical test method	Statistical test	NN–CART	NN–SVM
Wilcoxon test	Z	−1.9335	−2.0280
	one-sided pr <Z	0.0266	0.2130
	two-sided pr <|Z|	0.0532*	0.0426**
Kruskal–Wallis test	Chi square	4.1654	4.5570
	DF	1	1
	Pr >Chi square	0.0413**	0.0328**

* Significant at P < 0.1; ** significant at P < 0.05, *** significant at P < 0.01

## Conclusions

Certified public accountants (CPAs) and auditors check firms’ financial statements and issue their audit opinions and audit reports. These audit opinions and audit reports are very important for enterprises, stakeholders, and financial markets, especially investors. Thus, it is necessary to establish more accurate going concern doubt prediction models. The purpose of this study is to set up rigorous and reliable going concern doubt prediction models for auditors. This study applies the least absolute shrinkage and selection operator (LASSO) and data mining techniques (NN, CART, and SVM) to establish the prediction models.

According to the empirical results, the prediction accuracy is 88.96 % for the LASSO–NN model, is 88.75 % for the LASSO–CART model, and is 89.79 % for the LASSO–SVM model. This study uses LASSO to select important variables, which include: X4 (Debt ratio), X6 (Undistributed surplus), X20 (Total assets turnover), and X22 (Return on assets; ROA). As such, a firm’s top management, CPAs, and auditors all should pay close attention to them.

Type I errors may not have serious consequences when compared to Type II errors. If the auditor wrongly classifies a GC firm as healthy, then he/she can be sued. If an auditor issues a wrong audit report due to his/her misjudgment, then this will affect not only the enterprise and stakeholders, but also many investors. Moreover, the CPA may be sued. The costs for Type II errors are thus rather severe. We have developed three GCD prediction models. In the LASSO–NN model, the Type I error rate is 12.22 % and the Type II error rate is 7.50 %; in the LASSO–CART model, the Type I error rate is 13.61 % and the Type II error rate is 14.17 %; and in the LASSO–SVM model, the Type I error rate is 10.00 % and the Type II error rate is 15.83 %. These error rates are all lower than 20 %, especially in the LASSO–NN model where the Type II error rate is only 7.50 %. This is a key contribution of this paper.

Finally, the empirical results of this study can provide a reference for enterprises’ top management, CPAs, auditors, and future studies.

## Limitations

There are several limitations in this study. 1. The size of the financial market in Taiwan is not as big when compared to China, the U.S., UK, EU, Japan, etc.); 2. The Taiwan government has strict control over the listed companies and the financial market. Thus, GCD listed companies are fewer. 3. If the GCD prediction models are used in countries other than Taiwan, then the GCD indicators (variables) should be measured according to national or economically regional audit laws and regulations and financial practice.
